# Retinal amyloid angiopathy

**DOI:** 10.1590/0004-282X-ANP-2021-0297

**Published:** 2021-11-30

**Authors:** Alex Tiburtino Meira, Mario Teruo Sato, Naoye Shiokawa, Hélio Afonso Ghizoni Teive

**Affiliations:** 1 Universidade Federal da Paraíba, Departamento de Medicina Interna, Serviço de Neurologia, João Pessoa PB, Brazil. Universidade Federal da Paraíba Departamento de Medicina Interna Serviço de Neurologia João Pessoa PB Brazil; 2 Universidade Federal do Paraná, Departamento de Oftalmologia, Serviço de Oftalmologia, Curitiba PR, Brazil. Universidade Federal do Paraná Departamento de Oftalmologia Serviço de Oftalmologia Curitiba PR Brazil; 3 Universidade Federal do Paraná, Departamento de Medicina Interna, Serviço de Neurologia, Curitiba PR, Brazil. Universidade Federal do Paraná Departamento de Medicina Interna Serviço de Neurologia Curitiba PR Brazil

**Figure 1.**
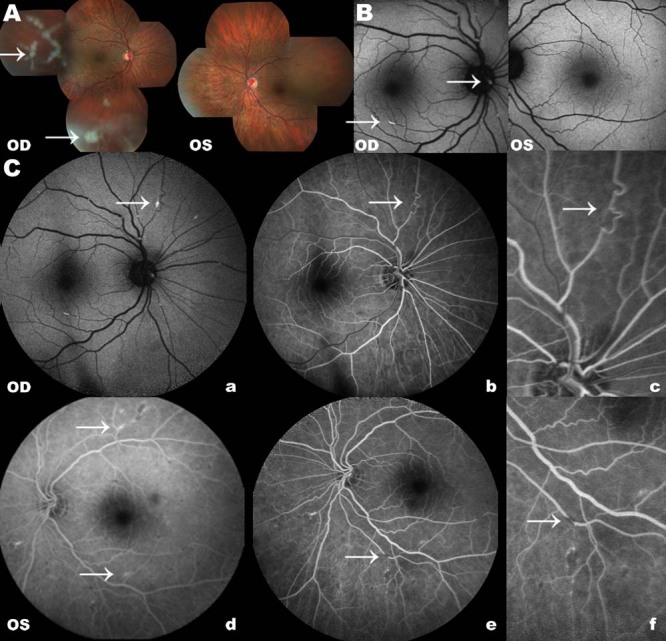
A: Retinography showing vitreous opacity in temporal periphery in OD. It was normal in the OS. B: FAF images show hyper autofluorescence whitish deposit above the optic disk and in the superior and inferior vascular arcades of the OD. The FAF was normal in the OS. C: Fluorescein angiography: a -whitish deposit in the nasal superior retina; b -superior peripheral vascular tortuosity, collateral secondary to arterial occlusion; c -detail of figure b; d -whitish deposit in the superior and inferior (hyper autofluorescence) temporal vascular arcades; e -arterial retinal occlusion in the inferior periphery in the OS; and f -detail of figure e.

OD: right eye; OS: left eye; FAF: fundus autofluorescence; arrow: alterations.

A 57-year-old male with previous myocardiopathy, polyneuropathy, bilateral cataract, and autonomic dysfunction had a family history of Familial Amyloid Polyneuropathy (FAP)^1^^,^^2^. When he was 55 years old, he underwent a genetic testing, which detected a deleterious heterozygous mutation c.325G>A, Glu109Lys, on exon 3 of the Transthyretin gene, for the diagnosis of FAP^3^. He was treated with Vyndaqel® (tafamidis meglumine) and amiodarone. He developed reduction of visual acuity in the right eye and floaters. Physical examination found that he had visual acuity of 20/40 (OD) and 20/25 (OS), with altered campimetry only in OD. The neuro-ophthalmological evaluation is provided in Figures 1-4. Laboratory testing excluded other hematological abnormalities. The final diagnosis was retinal amyloid angiopathy secondary to FAP. Ocular manifestations in FAP are rare; nevertheless, neurologists should investigate visual symptoms in patients with FAP. Retinal amyloid angiopathy is even rarer, but is a sight-threatening complication. Neurologists should assess the visual acuity, the intraocular pressure, vessel tortuosity, collaterals, or scalloped pupils, promptly indicating an ophthalmological evaluation^4^. Recent progress in the neuro-ophthalmological evaluation indicated that retinal amyloid angiopathy is more frequent than previously reported^4^_._ Therefore, neurologists should be aware of this complication in patients with FAP, especially in those presenting vitreous amyloidosis or longer duration of the disease^5^.
